# Supplementary material for: Clinical and Molecular Features in Medulloblastomas Subtypes in Children in a Cohort in Taiwan
Source: Cancers (Basel). 2022 Nov 3;14(21):5419. doi: 10.3390/cancers14215419 (PMC9657873; doi:10.3390/cancers14215419)
Supplement: Supplementary file 1 [file cancers-14-05419-s001.zip › cancers-1985217-supplementary.pdf]

Clinical and molecular features in medulloblastomas subtypes in children in a cohort in Taiwan

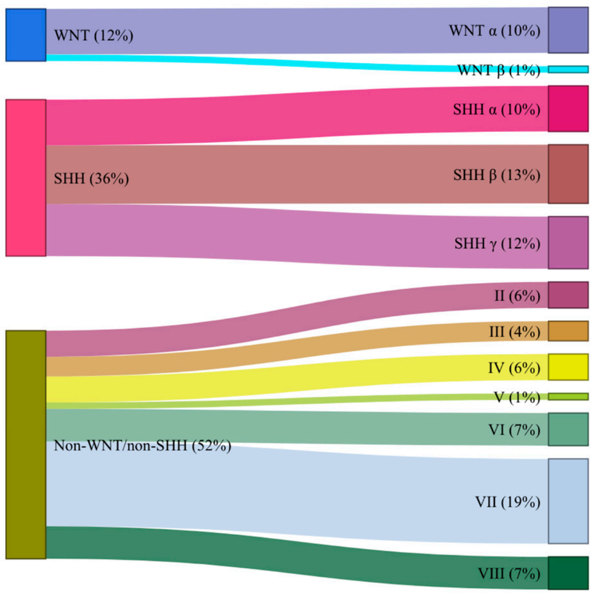

Figure S1. The clustering and relationship between subgroups and subtypes of MBs in this cohort.

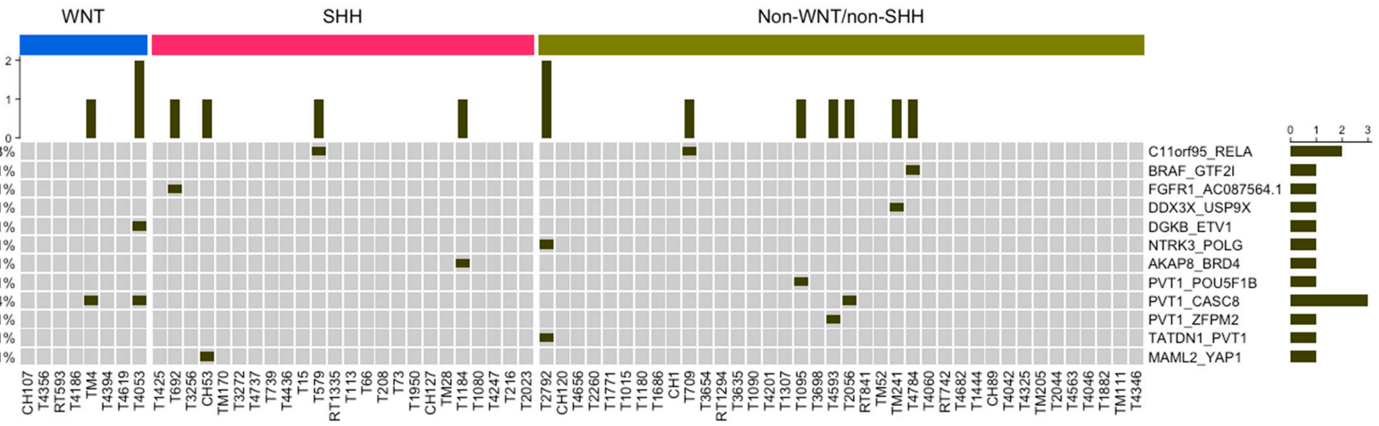

Figure S2. Fusion genes distributions in MB subgroups.

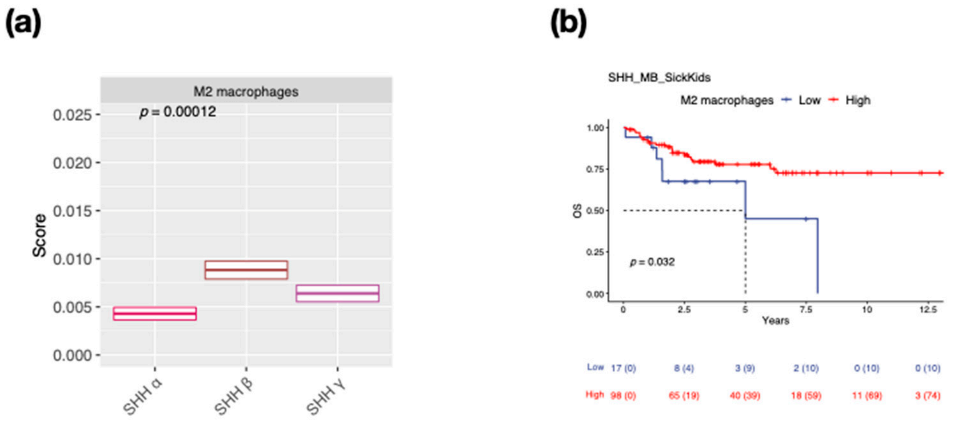

Figure S3. (a) The scores of the infiltrating M2 macrophages cells in SHH MB subtypes in the SickKids cohort. *p* value calculated by Kruskal-Wallis test. (b) The overall survival based on high or low M2 macrophages cells infiltration in SHH MBs in the SickKids cohort. *p* value calculated by log-rank test.

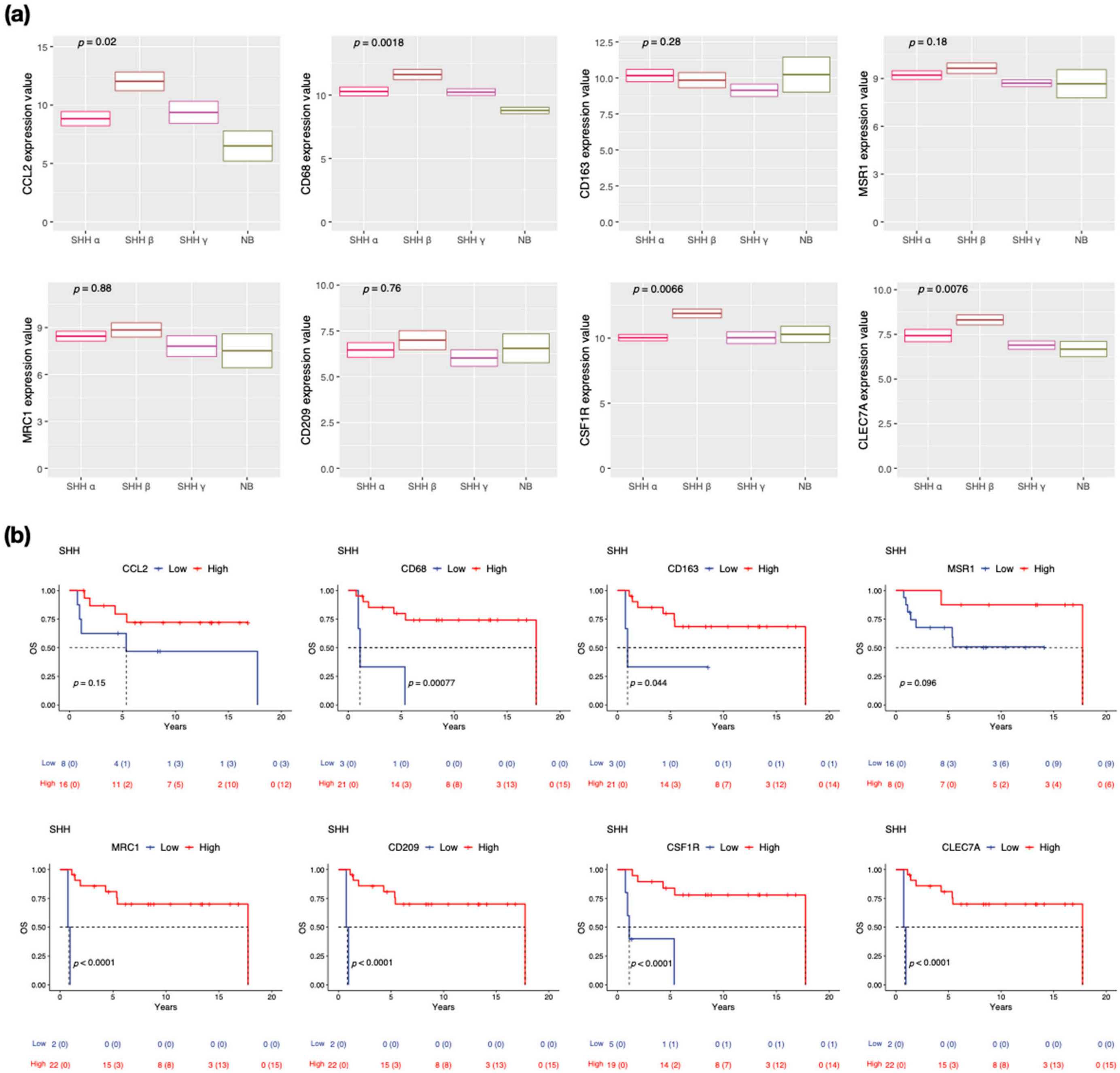

Figure S4. (a) The expression of CCL2, CD68, CD163, MSR1, MRC1, CD209, CSF1R, and CLEC7A in SHH MB subtypes. *p* value calculated by Kruskal-Wallis test. (b) The overall survival based on the expression of CCL2, CD68, CD163, MSR1, MRC1, CD209, CSF1R, and CLEC7A in SHH MBs. *p* value calculated by log-rank test.

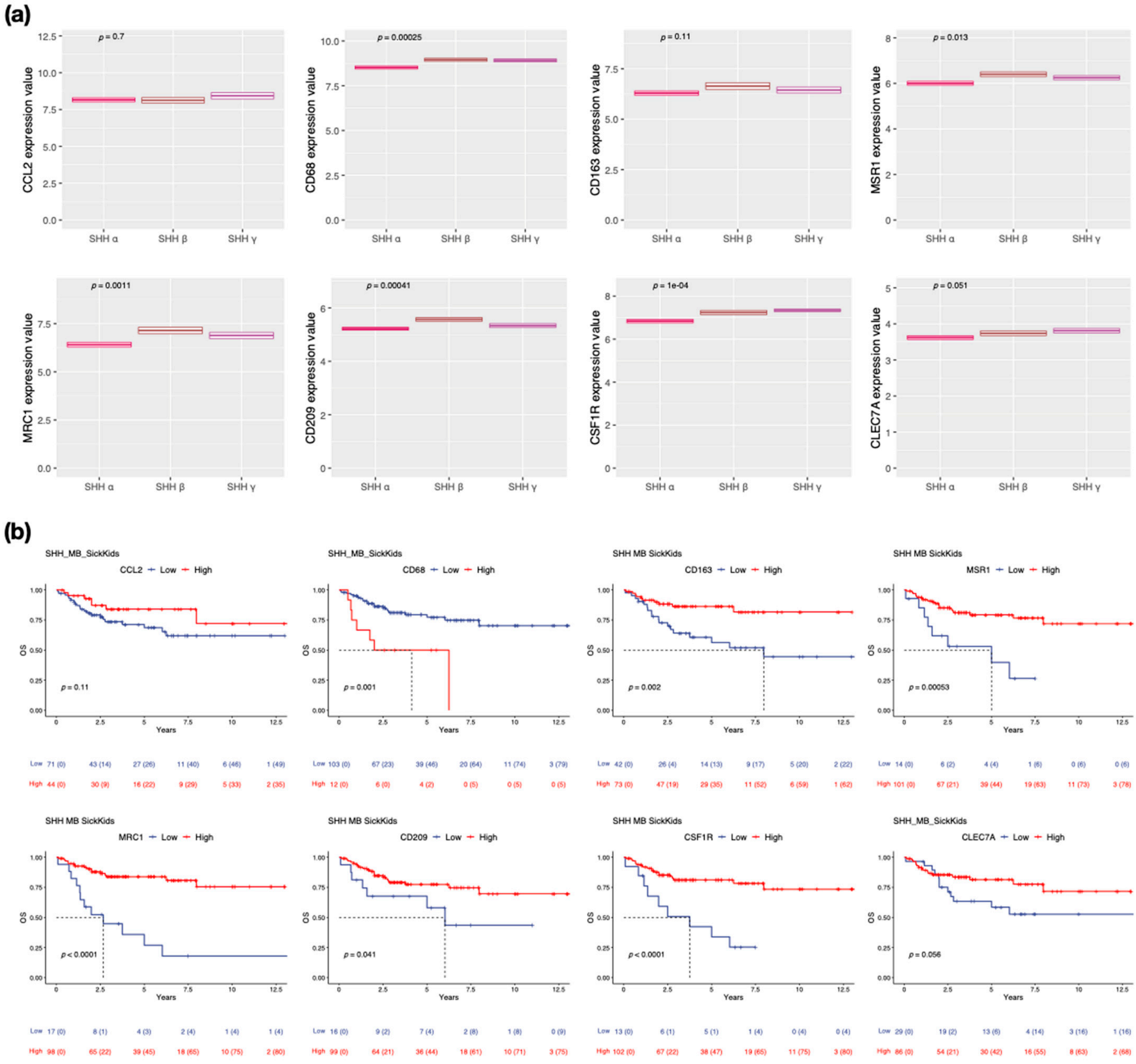

Figure S5. (a) The expression of CCL2, CD68, CD163, MSR1, MRC1, CD209, CSF1R, and CLEC7A in SHH MB subtypes in the SickKids cohort. *p* value calculated by Kruskal-Wallis test. (b) The overall survival based on the expression of CCL2, CD68, CD163, MSR1, MRC1, CD209, CSF1R, and CLEC7A in SHH MBs in the SickKids cohort. *p* value calculated by log-rank test.

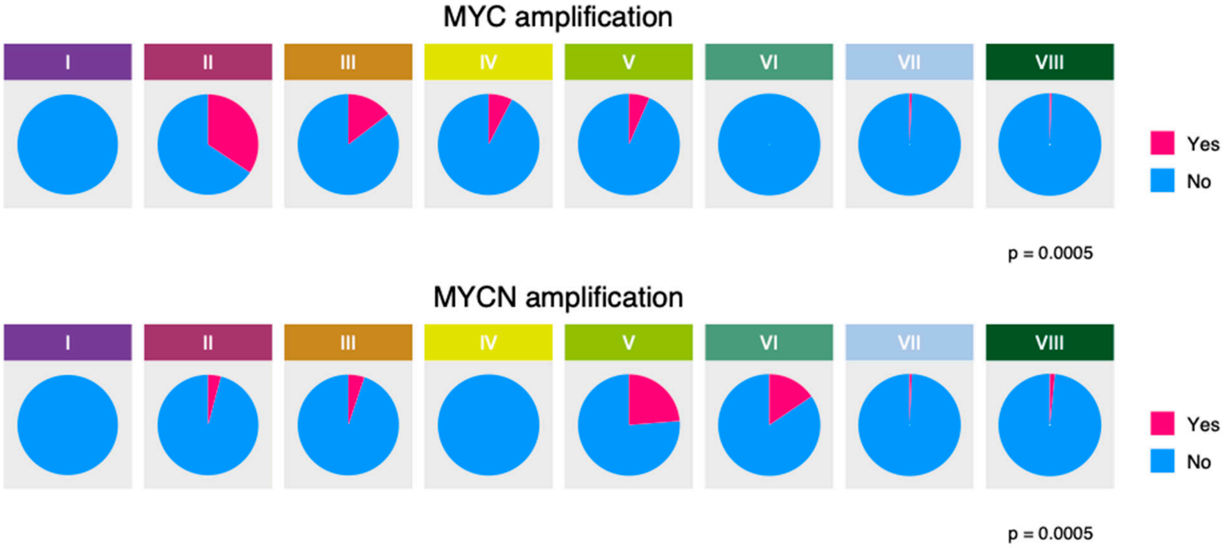

Figure S6. Distribution of focal MYC and MYCN events in non-WNT/non-SHH subtypes in Northcott et al. cohort.

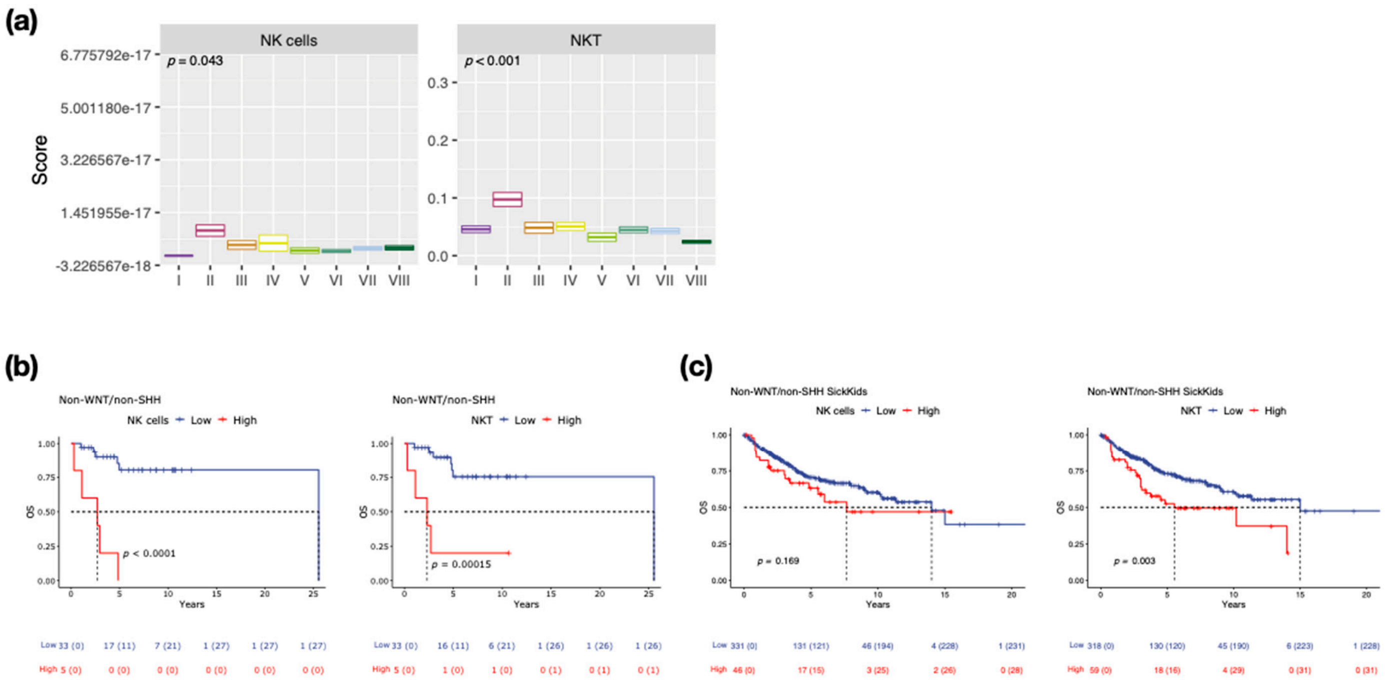

Figure S7. (a) The scores of the infiltrating NK and NKT cells in non-WNT/non-SHH MB subtypes in the SickKids cohort. *p* value calculated by Kruskal-Wallis test. The overall survival based on high or low NK or NKT cells infiltration in non-WNT/non-SHH MBs in our (b) and SickKids cohort (b). *p* value calculated by log-rank test.

Table S1. Demography and clinical annotations of molecular subtypes in our cohort series of 70 childhood Medulloblastomas (MBs) in Taiwan.

N = 70	WNT, n = 8 (11.4%)		SHH, n = 24 (32.7%)			Non-WNT/non-SHH, n = 38 (54.3%)						
	α	β	α	β	γ	II	III	IV	V	VI	VII	VIII
	7	1	7	9	8	4	3	4	1	5	13	5
**Frozen tissue for molecular profiling**												
Primary tumor	7	1	6	9	8	3	3	4	1	5	11	4
Recurrent tumor	0	0	1	0	0	1	0	0	0	0	2	1
**Age (median, range) at diagnosis (years)**												
7.2 (0.3–18.2)	7.9 (3.1–11.4)		4.1 (0.3–14.3)			6.9 (1.5–18.3)						
	8.4 (4.0-11.4)	3.1	6.5 (2.8–12.6)	4.1 (3.5–14.3)	1.4 (0.3–5.8)	5.5 (1.5-9.1)	4.3 (3.2-18.2)	3.0 (2.4-4.0)	15.4	6.6 (5.1-11.0)	8.3 (2.4-18.3)	10.1 (6.0-11.0)
**Age distribution (%)**												
≤3y, n = 13 (18.6)	0	0	1 (14.3)	0	7 (87.5)	1 (25.0)	0	2 (50.0)	0	0	1 (7.7)	0
>3y, n = 57 (81.4)	7 (100)	1	6 (85.7)	9 (100)	1 (12.5)	3 (75.0)	3 (100)	2 (50.0)	1	5 (100)	12 (92.3)	5 (100)
**Sex**												
Male, n = 35 (50%)	1	0	4	4	4	1	3	3	1	4	8	2
Female, n = 35 (50%)	6	1	3	5	4	3	0	1	0	1	5	3
Male/female ratio (1/1)	0.2/1		1.3/1	0.8/1	1/1	0.3/1		3/1		4/1	1.6/1	0.7/1
**Metastasis stage at diagnosis (M0-1, M2-3), number of cases (%)**												
M0-1, n = 50 (71.4)	7 (100)	1 (100)	6 (85.7)	6 (66.7)	8 (100)	2 (50.0)	2 (66.7)	2 (50.0)	1 (100)	3 (60.0)	8 (61.5)	4 (80.0)
M2-3, n = 20 (28.6)	0	0	1 (14.3)	3 (33.3)	0	2 (50.0)	1 (33.3)	2 (50.0)	0	2 (40.0)	5 (38.5)	1 (20.0)
**Extent of resection (NTR-TR, residue tumor<1.5 cm²; STR, residue tumor≥1.5 cm²), number of cases (%)**												
NTR-TR, n = 40 (57.1)	5 (71.4)	1 (100)	4 (57.1)	7 (77.8)	5 (62.5)	0	3 (100)	2 (50.0)	1 (100)	3 (60.0)	6 (46.2)	2 (40.0)
STR, n = 30 (42.9)	2 (28.6)	0	3 (42.9)	2 (22.2)	3 (37.5)	4 (100)	0	2 (50.0)	0	2 (40.0)	7 (53.8)	3 (60.0)
**Pathology variant, number of cases (%)**												
Classic, n = 34 (48.6)	4 (57.1)	1 (100)	2 (28.6)	7 (77.8)	3 (37.5)	1 (25.0)	1 (33.3)	3 (75.0)	0	3 (60.0)	3 (23.1)	4 (80.0)
DNMB, n = 11 (15.7)	1 (14.3)	0	3 (42.9)	2 (22.2)	4 (50.0)	0	0	0	0	0	1 (7.7)	0
MBEN, n = 1 (1.4)	0	0	0	0	1 (12.5)	0	0	0	0	0	0	0
LCA, n = 23 (32.9)	2 (28.6)	0	2 (28.6)	0	0	3 (75.0)	1 (33.3)	1 (25.0)	1 (100)	2 (40.0)	9 (69.2)	1 (20.0)
MMMB, n = 1 (1.4)	0	0	0	0	0	0	1 (33.3)	0	0	0	0	0
**Molecular subgroup-based clinical risk stratification, number of cases**												
Non-met (M0-1) AR, n = 34	7	1	4	6	5	0	2	1	1	2	3	2
Non-met (M0-1) HR, n = 16	0	0	2	0	3	2	0	1	0	1	5	2
Met (M2-3) HR, n =	0	0	1	3	0	2	1	2	0	2	5	1

20												
**Treatment strategy, number of cases (%)**												
CMT alone, n = 4 (5.7)	0	0	0	0	3 (37.5)	0	1 (33.3)	0	0	0	0	0
RT alone, n = 5 (7.1)	0	0	1 (14.3)	0	1 (12.5)	1 (25.0)	0	0	0	0	1 (7.7)	0
RT+CMT, n = 60 (85.7)	7 (100)	1 (100)	6 (85.7)	9 (100)	4 (50.0)	3 (75.0)	2 (66.7)	3 (75.0)	1 (100)	5 (100)	12 (92.3)	5 (100)
HDT/ SCR, n = 1 (1.4)	0	0	0	0	0	0	0	1 (25.0)	0	0	0	0
**Recurrence, number of cases (%)**												
Recurrence, n = 27 (38.6)	1 (14.3)	0	2 (28.6)	5 (55.6)	4 (50.0)	4 (100)	2 (66.7)	1 (25.0)	0	1 (20.0)	3 (23.1)	2 (40.0)
**Time from diagnosis to the first recurrence (years)**												
Median time (range)	1.5		0.8 (0.7-0.9)	2.6 (0.9-16.3)	0.7 (0.3-2.3)	1 (0.2-2.5)	1.4 (0.6-2.2)	0.9		1.5	1.9 (1.4-2.3)	10.4 (2.1-18.8)
**Median follow-up time (range) (years)**												
5.1 (0.3–25.5)	4.9 (2.8-11.7)	5.6	5.4 (1.1-16.8)	12.3 (1.9–17.7)	5.7 (0.7–13.4)	1.1 (0.3–4.8)	8.8 (1.1-9.6)	9.7 (2.5-11.5)	3.7	2.6 (1.4-7.4)	5.2 (2.5-12.5)	5.9 (1.8-25.5)
**Survivals of molecular subgroup (%)**												
5-year OS rate: 74.1	100	100	85.7	77.8	62.5	0	100	100		50.0	71.8	100
5-year RFS rate: 63	85.7	100	71.4	66.7	50.0	0	33.3	75.0		50.0	65.9	75.0

TR: Total resection, NTR: Near total resection, STR: Subtotal tumor resection, DNMB: Desmoplastic/nodular medulloblastoma, MBEN: Medulloblastoma with extensive nodularity, LCA: Large-cell/anaplastic, MMMB: Medulloblastoma with melanotic myogenic differentiation, Non-met: Nonmetastasis, Met: metastasis, M0-1: no metastasis to presence of tumor cells in CSF, M2: intracranial subarachnoid space or intracranial compartment metastasis, M3: intraspinal subarachnoid space metastasis, AR: average-risk, HR: high-risk, CMT: Chemotherapy, RT: Radiotherapy, HDT/SCR: high-dose therapy with stem-cell rescue, OS: Overall survival, RFS: Relapse-free survival.

Table S2. Comparison of demographic and clinical annotations of SHH MB subtypes in our and SickKids cohorts.

SHH						
Subtype	**α**		β		γ	
Study cohort	Our	SickKids	Our	SickKids	Our	SickKids
Frequency (%)	29.2	44.2	37.5	23.8	33.3	32.0
Age (median, range) at diagnosis (years)	6.5, 2.8–12.6	8.0, 2.0-45.0	4.1, 3.5–14.3	1.9, 0.2-6.0	1.4, 0.3–5.8	1.3, 0.5-41.7
Male/female ratio	1.3	1.7	0.8	0.9	1.0	1.2
Pathology variant (%)						
Classic	28.6	48.1	77.8	40.0	37.5	20.9
DNMB	42.9	28.8	22.2	53.3	50.0	53.5
MBEN	0	0	0	0	12.5	20.9
LCA	28.6	23.1	0	6.67	0	4.7
Metastasis (%)	14.3	20.0	33.3	33.3	0	9.4
5-year OS (%)	85.7	69.8	77.8	67.3	62.5	88.0

DNMB: Desmoplastic/nodular medulloblastoma, MBEN: Medulloblastoma with extensive nodularity, LCA: Large-cell/anaplastic, OS: Overall survival.

Table S3. Comparison of demographic and clinical annotations of non-WNT/non-SHH MB subtypes in our and SickKids cohorts.

Non-WNT/non-SHH																
Subtype	**I**		**II**		**III**		**IV**		**V**		**VI**		**VII**		**VIII**	
Study cohort	Our	SickKids	Our	SickKids	Our	SickKids	Our	SickKids	Our	SickKids	Our	SickKids	Our	SickKids	Our	SickKids
Frequency (%)	0	1.9	11.4	11.8	8.6	6.8	11.4	7.4	2.9	5.6	14.3	9.6	37.1	28.9	14.3	28.0
Age (median, range) at diagnosis (years)		6.5, 4.0-46.5	5.5, 1.5-9.1	4.7, 2.0-13.0	4.3, 3.2-18.2	4.9, 2.0-49.6	3.0, 2.4-4.0	3.6, 1.2-8.2	15.4	9.0, 2.8-17.0	6.6, 5.1-11.0	7, 2.5-18.0	8.3, 2.4-18.3	8.0, 1.0-21.0	10.1, 6.0-11.0	10.0, 4.0-48.2
Male/female ratio		0.7	0.3	2.5		10.0	3.0	3.2		4.7	4	1.6	1.6	1.7	0.7	3.0
Pathology variant (%)																
Classic		75.0	25.0	52.4	33.3	80.0	75.0	68.8	0	50.0	60.0	69.6	23.1	80.5	80.0	93.6
DNMB		0	0	4.8	0	0	0	18.8	0	33.3	0	8.7	7.7	10.4	0	4.8
MBEN		0	0	4.8	0	0	0	0	0	8.3	0	4.3	0	2.6	0	1.6
LCA		25.0	75.0	38.1	33.3	20.0	25.0	12.5	100	8.3	40.0	17.4	69.2	6.5	20.0	0
MMMB		0	0	0	33.3	0	0	0	0	0	0	0	0	0	0	0
Metastasis (%)		0	50.0	52.0	33.3	38.9	50.0	58.8	0	46.7	40.0	30.4	38.5	36.1	20.0	40.3
5-year OS (%)		100	0	47.6	100	36.5	100	93.3		70.7	50.0	92.3	71.8	79.1	100	78.2

DNMB: Desmoplastic/nodular medulloblastoma, MMMB: Medulloblastoma with melanotic myogenic differentiation, LCA: Large-cell/anaplastic, OS: Overall survival.
